# Morbidity Risk of Infectious Gastroenteritis Attributable to Cold and Heat Temperatures: An Analysis of National Surveillance Data in Japan (2000-2019)

**DOI:** 10.7759/cureus.76367

**Published:** 2024-12-25

**Authors:** Keita Wagatsuma

**Affiliations:** 1 Epidemiology and Public Health, Graduate School of Medical and Dental Sciences, Niigata University, Niigata, JPN

**Keywords:** ambient temperature, climate variability, infectious gastroenteritis, japan, seasonality

## Abstract

Introduction

Climate change is a decisive factor affecting human health. While many epidemiological studies have investigated the acute impacts of ambient temperature on mortality and morbidity, the global burden of infectious gastroenteritis linked to temperature changes remains largely unexplored. Therefore, we aimed to examine the exposure-response associations between ambient temperature and infectious gastroenteritis incidence throughout Japan and quantify the temperature-related morbidity burden.

Methods

Weekly time-series data from 2000 to 2019, encompassing meteorological factors and infectious gastroenteritis cases, were collected from all 47 Japanese prefectures. A two-stage time-series design was employed. In the first stage, quasi-Poisson regression models combined with distributed lag non-linear models were used for each prefecture. In the second stage, a multivariate meta-analysis was conducted to derive national estimates. The attributable fractions were determined for both low and high temperatures, categorized as temperatures below or above the minimum risk temperature, respectively.

Results

The analysis included 19,571,939 infectious gastroenteritis cases. The exposure-response association between temperature and infectious gastroenteritis cases was non-linear, exhibiting an approximate M-shaped relationship. Overall, 51.7% (95% empirical confidence interval (eCI): 42.6, 58.4) of infectious gastroenteritis cases were attributable to non-optimal temperatures in Japan. The attributable fraction to low temperatures was 47.6% (95% eCI: 38.5, 54.2), whereas that of high temperatures was 4.1% (95% eCI: 2.4, 5.5).

Conclusion

The majority of the temperature-related infectious gastroenteritis burden in Japan was attributable to lower temperatures. Our findings indicate that public health strategies aimed at mitigating the burden of infectious gastroenteritis should take temperature levels into account.

## Introduction

Infectious gastroenteritis is a prevalent disease worldwide, presenting symptoms of vomiting and diarrhea, and is caused by various pathogens, including viruses, bacteria, and parasites [1]. The incidence of infectious gastroenteritis exhibits seasonality, marked by periodic fluctuations aligned with seasons or specific calendar periods, similar to other infectious diseases [2]. Consequently, various epidemiological studies have examined the association between ambient temperature and infectious gastroenteritis, a relationship that has gained increased relevance in the context of climate change [3].

Most epidemiological studies have evaluated the association between meteorological variables and infectious gastroenteritis in terms of relative risk [4]. However, these indicators provide limited information on the excess burden due to exposure, such as relative excess measures of infectious gastroenteritis. The attributable fraction, which accounts for both exposure risk and the frequency of days when such risk occurs, serves as a key metric for assessing health burdens associated with exposure [5]. Nevertheless, limited studies have examined the risk of infectious gastroenteritis that is attributable to the range of temperatures nationwide. Furthermore, the optimal temperature thresholds that minimize temperature impacts on major disease categories remain unclear.

This study therefore investigates the morbidity risk of infectious gastroenteritis attributable to non-optimal temperatures, as well as the relative contributions of low and high temperatures, over a span of approximately 20 years across 47 prefectures of Japan, using national surveillance data. This study aims to achieve three primary objectives: (i) to quantify the proportion of infectious gastroenteritis cases attributable to non-optimal temperatures, (ii) to distinguish the differential effects of low and high temperatures on morbidity, and (iii) to evaluate regional disparities in the temperature-related morbidity burden across Japan. To the best of our knowledge, this is a valuable study that addresses this question and undertakes a comprehensive assessment of the Japanese population.

## Materials and methods

Data collection

National surveillance data on the weekly counts of newly confirmed infectious gastroenteritis cases from 2000 to 2019 were obtained from the National Institute of Infectious Diseases (NIID), Ministry of Health, Labour and Welfare, Japan [6]. These data encompass all 47 prefectures, covering the entire country. Infectious gastroenteritis was designated as a notifiable disease in 1999 and has since been systematically monitored under Japan's Infectious Disease Control Law. Approximately 3,000 pediatric sentinel sites, representing around 8.0% of all pediatric hospitals and clinics in Japan, contribute to this surveillance system. The case definition for infectious gastroenteritis includes clinical manifestations such as acute abdominal pain, vomiting, diarrhea, dehydration, fever, nausea, and electrolyte imbalances.

Data on daily mean ambient temperature (°C) and relative humidity (%) were sourced from representative monitoring stations in each prefecture, as reported by the Japan Meteorological Agency [7]. Weekly averages of these variables were calculated for the corresponding weeks from 2000 to 2019 for each prefecture.

Statistical modeling

A two-stage time-series meta-analysis was performed using data from 47 prefectures in Japan to examine the morbidity risk of infectious gastroenteritis attributable to ambient temperature [8].

In the first stage, the exposure-response relationship between weekly mean temperature and infectious gastroenteritis cases was analyzed for each prefecture using quasi-Poisson regression combined with distributed lag non-linear models. The cross-basis function of weekly mean temperature was constructed using a natural cubic B-spline function, with three internal knots positioned at the 10^th^, 75^th^, and 90^th^ percentiles of the temperature distribution specific to each prefecture. Based on previous studies, we examined lag periods of up to four weeks for mean temperature using a natural cubic B-spline function with three internal knots placed at equal distances in log scale distributions [9]. We also included a natural cubic B-spline of time with 8 degrees of freedom (df) per year to account for seasonal and long-term trends. We also included a natural cubic B-spline of mean relative humidity with three df, the week number of the year, and the number of national public holidays per week in the main model to remove the potential confounding effects. We also incorporated the autoregressive term of logged weekly counts at lags of one and two weeks. We reduced the dimension of the coefficients to be compatible with the multivariable meta-analysis in the second-stage modeling. Based on the lag-cumulative non-linear associations fitted in the model above, cold (5^th^) and heat (95^th^) risks were quantified as relative risks compared with the risk for the minimum morbidity temperature (MMT), where the minimum risk was identified between the 1^st^ and 99^th^ percentiles of temperature. The MMTs were re-centered based on the best linear unbiased predictions (BLUPs) described in the second-stage modeling.

In the second stage, we utilized a multivariate random-effects meta-regression analysis to pool the prefecture-specific estimates at the national level and estimate the BLUPs for the overall cumulative temperature-morbidity association [8]. To account for effect modification due to inter-prefecture differences, we incorporated meta-predictors, including prefecture-specific temperature, temperature range, and relative humidity.

Population-attributable risks for low and high temperatures were calculated by summing subsets corresponding to weeks with temperatures below or above the MMT [5]. The MMT is the temperature at which the morbidity risk is lowest. The 95% empirical confidence intervals (eCI) for attributable risks were derived using Monte Carlo simulations, generating 1,000 coefficient samples based on a multivariate normal distribution of the BLUPs and their associated (co)variance matrix.

Sensitivity analyses were conducted to evaluate the robustness of the modeling approach by adjusting for different df for seasonal and long-term trends (6 and 10 df per year), lag periods (six weeks), autocorrelation, and relative humidity across the 47 prefectures. Ethical approval was deemed unnecessary as the analysis relied solely on anonymized data. All statistical analyses were conducted in R version 4.1.0 (R Foundation for Statistical Computing, Vienna, Austria) using the “*dlnm*” and “*mixmeta*” packages.

## Results

Descriptive analysis

A total of 19,571,939 infectious gastroenteritis cases were included in the analysis (Table 1). The overall weekly mean temperature was 15.6°C, while prefecture-specific mean temperatures varied widely, ranging from 9.5°C in Hokkaido to 23.5°C in Okinawa.

**Table 1:** Descriptive statistics of newly confirmed infectious gastroenteritis cases (n = 19,571,939) and meteorological variables across 47 prefectures of Japan from 2000 to 2019. SD: standard deviation

Prefecture	Newly confirmed cases		Ambient temperature (℃)		Relative humidity (%)	
	Mean	SD	Mean	SD	Mean	SD
Hokkaido	392	217	9.5	9.4	68.5	6.9
Aomori	145	96	10.9	8.8	74.9	5.3
Iwate	171	116	10.9	9.2	73.8	7.3
Miyagi	422	311	13.1	8.1	70.9	9.3
Akita	162	110	12.4	8.9	72.9	6.1
Yamagata	207	156	12.3	9.2	73.7	8.5
Fukushima	251	175	13.7	8.6	69.1	8.6
Ibaraki	347	235	14.4	8.0	72.7	8.8
Tochigi	189	159	14.6	8.3	69.0	9.7
Gunma	355	253	15.3	8.3	61.5	10.6
Saitama	1128	788	15.8	8.2	64.0	11.3
Chiba	764	553	16.5	7.5	67.5	11.3
Tokyo	1266	998	16.8	7.7	62.3	12.2
Kanagawa	1248	888	16.5	7.4	66.6	11.4
Niigata	357	262	14.4	8.5	71.6	6.0
Toyama	219	144	14.8	8.6	76.4	7.6
Ishikawa	208	131	15.3	8.4	70.7	6.5
Fukui	213	128	15.1	8.7	74.6	7.2
Yamanashi	110	80	15.4	8.5	62.6	10.2
Nagano	346	208	12.5	9.3	71.9	7.6
Gifu	195	130	16.5	8.5	65.3	7.9
Shizuoka	560	359	17.2	7.2	68.0	10.4
Aichi	973	658	16.5	8.4	64.8	8.0
Mie	348	224	16.6	8.0	66.8	7.8
Shiga	169	110	15.3	8.5	73.6	5.4
Kyoto	425	232	16.5	8.5	65.9	5.7
Osaka	1142	635	17.4	8.1	63.4	6.6
Hyogo	907	496	17.3	8.0	65.1	7.0
Nara	177	117	15.5	8.3	72.4	6.9
Wakayama	147	106	17.2	7.9	65.4	7.0
Tottori	144	85	15.5	8.3	73.1	6.5
Shimane	159	86	15.5	8.1	75.1	6.2
Okayama	356	199	16.6	8.4	66.7	7.0
Hiroshima	485	293	16.7	8.2	66.3	7.0
Yamaguchi	363	243	16.0	8.2	72.0	6.8
Tokushima	140	83	17.1	7.8	67.2	8.0
Kagawa	192	118	17.0	8.2	66.2	7.4
Ehime	319	188	17.0	7.8	66.4	7.3
Kochi	150	117	17.6	7.5	69.2	8.9
Fukuoka	932	510	17.6	7.7	67.0	8.0
Saga	123	106	17.2	8.0	68.9	7.0
Nagasaki	234	169	17.6	7.4	70.4	8.0
Kumamoto	394	226	17.5	8.1	69.5	7.2
Oita	402	214	17.1	7.6	68.3	8.4
Miyazaki	388	223	18.0	7.1	73.6	8.4
Kagoshima	410	246	19.0	7.2	69.7	7.4
Okinawa	86	55	23.5	4.5	72.8	7.9
Nationwide	400	465	15.6	8.4	69.0	8.9

Exposure-response relationships

**Figure 1:**
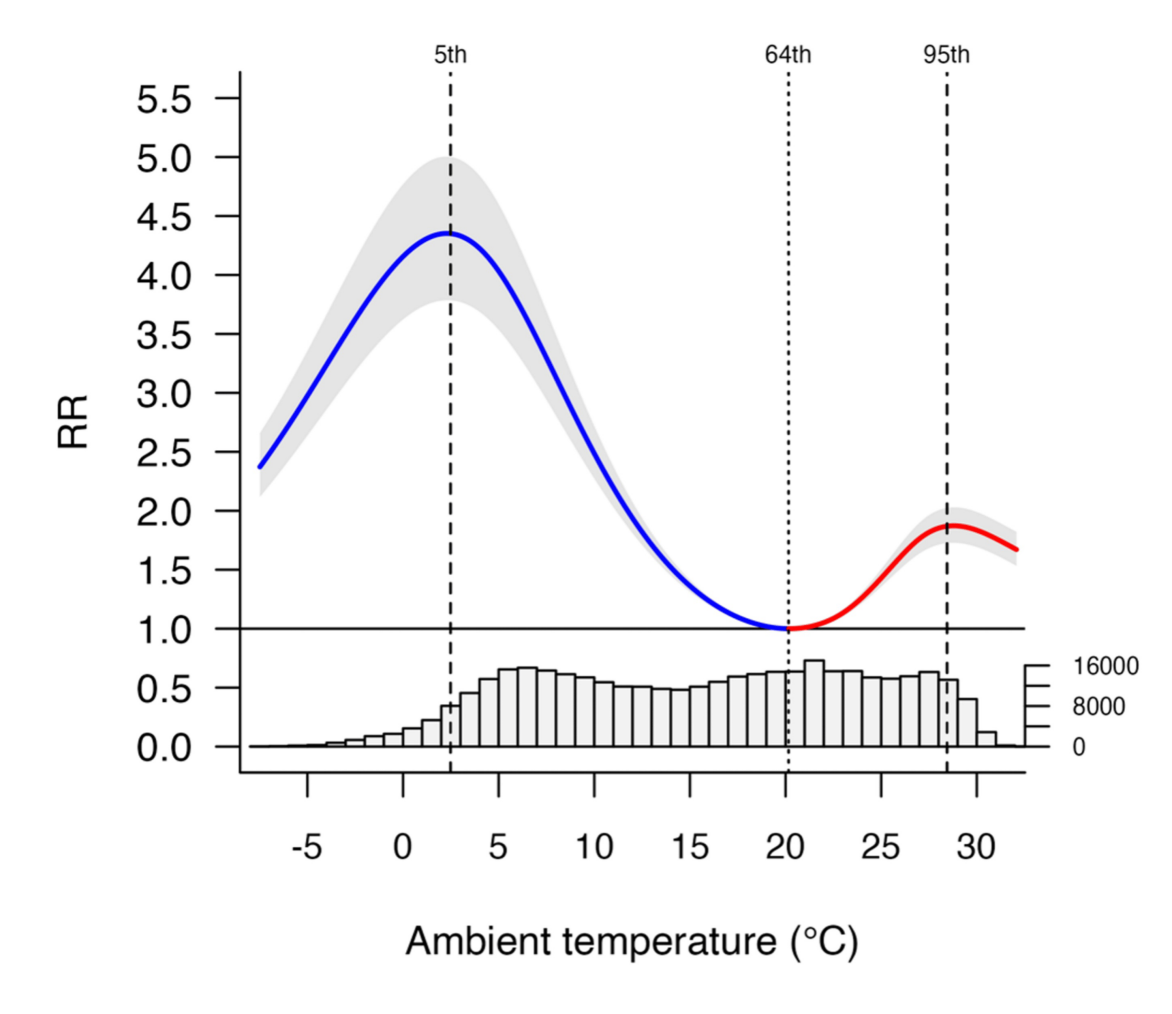
Overall cumulative exposure-response relationship between the relative risk (RR; 95% eCI) of infectious gastroenteritis incidence and ambient temperature in Japan. These associations were depicted as the best linear unbiased predictions, accompanied by shaded grey areas representing the 95% eCI. Solid grey lines marked the minimum morbidity temperatures (MMTs), while dashed lines indicated the 5^th^ and 95^th^ percentiles. RR: relative risk; eCI: empirical confidence interval

**Figure 2:**
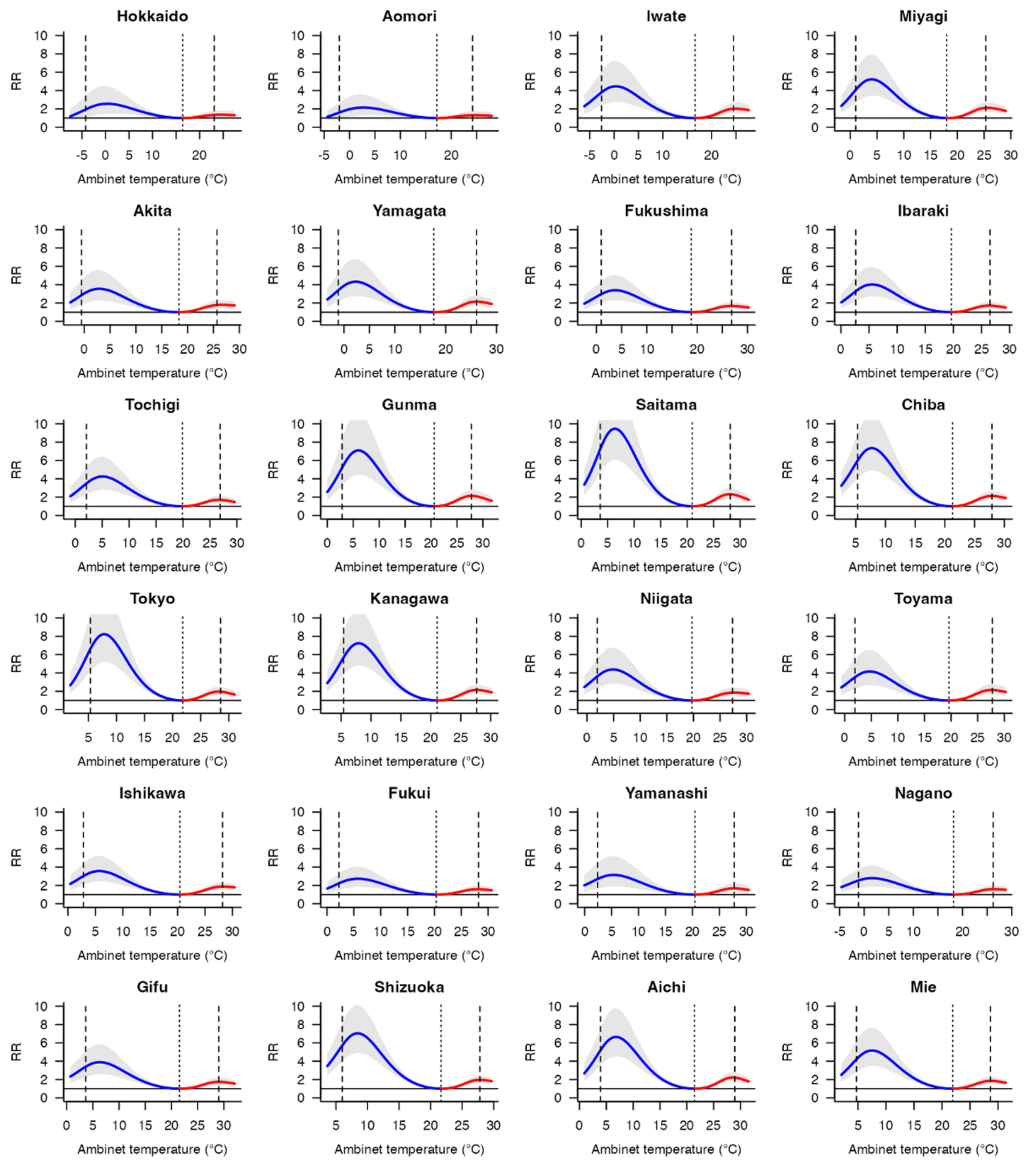
Overall cumulative exposure-response relationship between the relative risk (RR; 95% eCI) of infectious gastroenteritis incidence and ambient temperature across Japan's 47 prefectures. These associations were depicted as the best linear unbiased predictions, accompanied by shaded grey areas representing the 95% eCI. Solid grey lines marked the minimum morbidity temperatures (MMTs), while dashed lines indicated the 5^th^ and 95^th^ percentiles. RR: relative risk; eCI: empirical confidence interval

**Figure 3:**
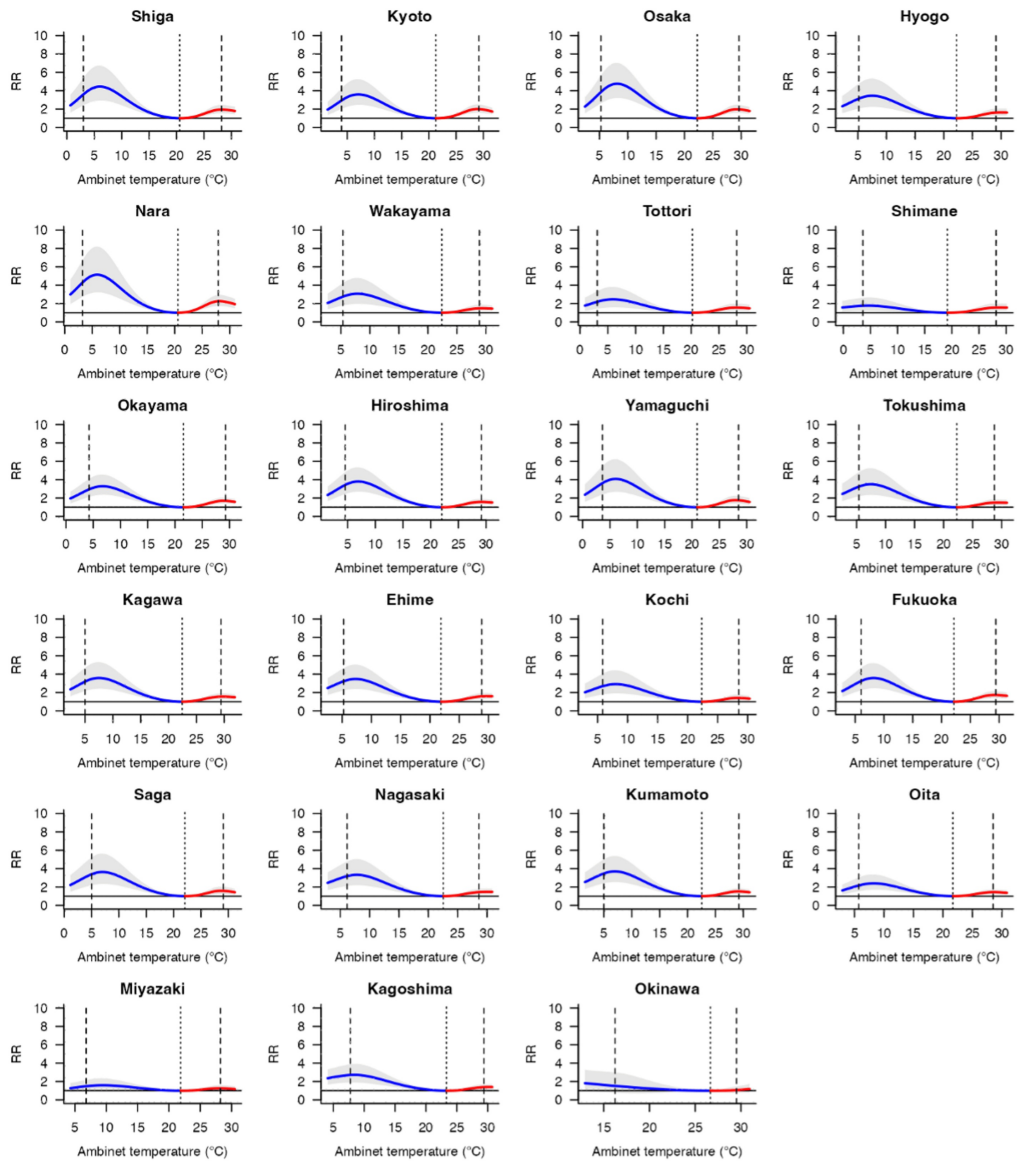
Overall cumulative exposure-response relationship between the relative risk (RR; 95% eCI) of infectious gastroenteritis incidence and ambient temperature across Japan's 47 prefectures. These associations were depicted as the best linear unbiased predictions, accompanied by shaded grey areas representing the 95% eCI. Solid grey lines marked the minimum morbidity temperatures (MMTs), while dashed lines indicated the 5^th^ and 95^th^ percentiles. RR: relative risk; eCI: empirical confidence interval

Figure 1 shows the overall cumulative exposure-response relationships between mean temperature and infectious gastroenteritis incidence in Japan. The risk of infectious gastroenteritis showed a non-linear bimodal peak at temperatures below and above MMT. Corresponding graphs for all 47 prefectures using BLUPs are included in Figures 2-3.

Attributable risk

Table 2 provides details on the attributable risks associated with low and high temperatures for each prefecture. At the national level, the MMT of the mean temperature corresponded to the 64^th^ percentile, with prefecture-specific MMTs ranging from the 63^rd^ percentile in Shimane to the 70^th^ percentile in Hokkaido and Aomori, reflecting regional climatic diversity. Overall, 51.7% (95% eCI: 42.6, 58.4) of infectious gastroenteritis cases were attributable to non-optimal temperatures. The majority of this burden was attributed to low temperatures, which accounted for 47.6% (95% eCI: 38.5, 54.2) of infectious gastroenteritis cases, while high temperatures contributed to 4.1% (95% eCI: 2.4, 5.5).

**Table 2:** Attributable fraction (95% eCI) for infectious gastroenteritis incidence by prefecture, expressed as total and separate components for low and high ambient temperatures. Details on the prefecture-specific minimum morbidity percentile (MMP), minimum morbidity temperature (MMT), and the proportion (%) of infectious gastroenteritis cases attributable to temperature are provided for each prefecture in Japan. The attributable fraction is presented as a total, along with its components attributable to low and high temperatures, with 95% empirical confidence intervals (eCIs) included.

Prefecture	MMP	MMT (°C)	Fraction attributable to non-optimal temperatures (%)	Fraction attributable to low temperature (%)	Fraction attributable to high temperature (%)
Hokkaido	70^th^	16.4	36.5 (18.0, 48.2)	34.3 (17.8, 46.6)	2.2 (-0.1, 4.2)
Aomori	70^th^	17.2	34.3 (11.5, 48.8)	32.5 (8.5, 46.8)	1.7 (-0.4, 3.4)
Iwate	66^th^	16.6	54.3 (42.7, 61.6)	49.3 (38.3, 56.7)	4.9 (3.2, 6.4)
Miyagi	67^th^	18.0	56.8 (49.3, 62.6)	52.5 (44.2, 58.5)	4.3 (2.9, 5.4)
Akita	68^th^	18.3	49.6 (36.9, 58.4)	45.7 (33.6, 54.5)	3.9 (2.0, 5.3)
Yamagata	65^th^	17.7	53.9 (44.1, 61.2)	49.5 (39.3, 56.4)	4.4 (2.8, 5.6)
Fukushima	66^th^	18.8	47.7 (37.9, 55.7)	44.4 (34.7, 52.1)	3.2 (1.8, 4.5)
Ibaraki	69^th^	19.6	51.7 (42.4, 58.2)	47.7 (38.6, 54.6)	3.9 (2.4, 5.3)
Tochigi	67^th^	19.9	55.2 (45.0, 61.6)	52.3 (42.0, 59.1)	2.8 (1.6, 3.9)
Gunma	67^th^	20.6	61.5 (54.5, 66.8)	56.5 (49.5, 61.6)	4.9 (3.4, 6.1)
Saitama	67^th^	21.0	64.6 (59.1, 68.4)	59.6 (54.6, 63.6)	5.0 (3.6, 6.1)
Chiba	69^th^	21.3	62.4 (56.7, 67.0)	58.2 (52.5, 62.6)	4.1 (3.0, 5.1)
Tokyo	70^th^	21.8	60.9 (54.0, 65.8)	56.4 (50.5, 61.0)	4.4 (3.0, 5.6)
Kanagawa	68^th^	21.1	61.4 (54.9, 66.0)	57.0 (50.8, 61.4)	4.4 (3.3, 5.4)
Niigata	67^th^	19.7	54.2 (44.4, 61.3)	51.1 (41.6, 57.8)	3.1 (1.9, 4.1)
Toyama	65^th^	19.6	51.8 (41.5, 59.3)	46.5 (35.9, 54.1)	5.3 (3.5, 6.7)
Ishikawa	67^th^	20.4	49.4 (39.7, 56.6)	45.3 (35.5, 52.8)	4.0 (2.6, 5.2)
Fukui	67^th^	20.3	41.5 (28.4, 50.5)	37.8 (25.8, 46.9)	3.7 (1.8, 5.3)
Yamanashi	66^th^	20.5	46.1 (30.2, 56.5)	42.5 (27.9, 52.7)	3.6 (1.4, 5.4)
Nagano	66^th^	18.2	41.9 (30.2, 50.3)	38.3 (27.1, 46.4)	3.5 (1.6, 5.1)
Gifu	66^th^	21.5	50.3 (40.8, 56.7)	46.4 (37.0, 53.3)	3.9 (2.2, 5.3)
Shizuoka	68^th^	21.7	60.0 (54.5, 64.2)	55.1 (50.0, 59.2)	4.9 (3.4, 6.1)
Aichi	66^th^	21.4	60.1 (53.7, 64.5)	54.7 (49.0, 59.5)	5.3 (4.1, 6.6)
Mie	69^th^	21.9	56.6 (49.2, 62.1)	52.1 (43.9, 57.6)	4.4 (3.0, 5.5)
Shiga	68^th^	20.6	53.0 (44.4, 59.6)	48.3 (38.8, 54.4)	4.7 (3.1, 6.1)
Kyoto	66^th^	21.3	47.4 (38.1, 53.7)	42.0 (33.5, 48.4)	5.3 (3.7, 6.7)
Osaka	67^th^	22.3	52.3 (45.1, 57.9)	47.1 (39.7, 53.1)	5.1 (3.6, 6.5)
Hyogo	67^th^	22.2	45.4 (34.4, 53.4)	41.8 (30.3, 48.9)	3.6 (1.7, 5.3)
Nara	67^th^	20.6	56.2 (46.6, 63.0)	51.2 (42.1, 57.5)	5.0 (3.3, 6.2)
Wakayama	69^th^	22.4	46.1 (33.7, 55.4)	43.5 (29.0, 52.7)	2.5 (0.8, 4.0)
Tottori	67^th^	20.2	37.9 (23.3, 48.9)	34.1 (19.6, 43.7)	3.7 (1.5, 5.6)
Shimane	63^rd^	19.2	27.1 (10.0, 39.3)	22.6 (5.4, 34.1)	4.4 (1.6, 6.5)
Okayama	66^th^	21.6	46.0 (38.2, 52.4)	41.3 (33.0, 47.8)	4.7 (2.9, 6.0)
Hiroshima	68^th^	22.0	50.1 (42.6, 56.3)	46.7 (38.7, 52.8)	3.3 (1.8, 4.5)
Yamaguchi	66^th^	20.9	51.6 (42.2, 58.7)	47.6 (37.6, 54.8)	4.0 (2.4, 5.4)
Tokushima	69^th^	22.3	47.5 (37.7, 55.1)	44.2 (33.9, 51.9)	3.3 (1.5, 4.9)
Kagawa	69^th^	22.4	48.2 (38.3, 55.6)	44.9 (34.9, 51.8)	3.3 (1.6, 4.6)
Ehime	68^th^	21.9	47.3 (38.1, 54.0)	43.9 (34.5, 50.7)	3.3 (1.7, 4.8)
Kochi	68^th^	22.4	45.1 (32.1, 54.7)	42.7 (29.8, 52.1)	2.3 (0.7, 3.7)
Fukuoka	67^th^	22.1	47.1 (38.4, 53.9)	42.8 (33.6, 49.5)	4.2 (2.6, 5.7)
Saga	66^th^	22.1	51.4 (39.1, 60.3)	49.2 (37.6, 57.7)	2.1 (0.8, 3.2)
Nagasaki	69^th^	22.5	46.5 (36.5, 54.8)	44.1 (32.6, 52.3)	2.3 (0.7, 3.7)
Kumamoto	67^th^	22.6	47.3 (38.6, 54.3)	43.8 (35.3, 50.3)	3.4 (1.4, 5.0)
Oita	68^th^	21.7	37.3 (26.5, 45.1)	33.9 (22.7, 42.7)	3.3 (1.3, 5.0)
Miyazaki	66^th^	21.9	23.1 (5.7, 35.6)	20.8 (1.6, 33.2)	2.3 (-0.3, 4.3)
Kagoshima	67^th^	23.3	38.7 (27.0, 47.6)	35.6 (25.2, 43.8)	3.0 (0.9, 4.9)
Okinawa	68^th^	26.7	13.9 (-23.2, 33.2)	13.0 (-19.1, 31.7)	0.9 (-4.7, 5.7)
Nationwide	64^th^	20.1	51.7 (42.6, 58.4)	47.6 (38.5, 54.2)	4.1 (2.4, 5.5)

Sensitivity analysis confirmed that altering the model specifications had minimal impact on the estimates (Table 3).

**Table 3:** Sensitivity analysis using the fraction (with 95% empirical confidence interval (eCI)) attributable to ambient temperature (total, high and low temperature components) by varying the modeling choices and controlling for different degrees of freedom (df) for the seasonal and long-term trends (6 and 10 df per year), lag periods (six weeks), autocorrelation, and relative humidity. Df: degrees of freedom

	Fraction attributable to non-optimal temperatures (%)	Fraction attributable to low temperature (%)	Fraction attributable to high temperature (%)
Modeling choices (47 prefectures)			
Main model	51.7 (42.6, 58.4)	47.6 (38.5, 54.2)	4.1 (2.4, 5.5)
Df per year for seasonal control: 6	32.1 (14.9, 44.6)	31.8 (14.9, 44.3)	0.3 (0.0, 0.6)
Df per year for seasonal control: 10	48.4 (33.3, 59.2)	48.4 (33.5, 59.1)	0.0 (-0.1, 0.1)
Lag periods: 6 weeks	57.9 (46.3, 65.0)	53.9 (42.2, 61.2)	4.0 (1.4, 5.9)
Control for autocorrelation (47 prefectures)			
With autocorrelation (main model)	51.7 (42.6, 58.4)	47.6 (38.5, 54.2)	4.1 (2.4, 5.5)
Without autocorrelation	52.7 (43.2, 59.4)	48.5 (38.9, 55.2)	4.2 (2.6, 5.6)
Control for relative humidity (47 prefectures)			
With relative humidity (main model)	51.7 (42.6, 58.4)	47.6 (38.5, 54.2)	4.1 (2.4, 5.5)
Without relative humidity	51.5 (42.4, 58.2)	47.6 (38.6, 54.0)	3.9 (2.4, 5.3)

## Discussion

Our comprehensive nationwide study revealed several significant findings. Notably, we found that exposure to ambient temperature accounted for a considerable portion of the infectious gastroenteritis burden, with low ambient temperatures contributing most to the morbidity burden in Japan. Additionally, we also identified variations in ambient temperatures associated with minimum morbidity due to infectious gastroenteritis across the 47 Japanese prefectures examined.

Our analysis indicated that 51.7% of infectious gastroenteritis cases were attributable to non-optimal ambient temperatures. The observed exposure-response curves may be attributed to the etiological agents responsible for infectious gastroenteritis cases. Previous epidemiological evidence consistently demonstrates that low temperatures increase the risk of viral gastroenteritis, aligning with this study's findings. Indeed, a systematic review of 29 studies on norovirus reported that 71.0% of outbreaks occurred during winter in the Northern Hemisphere [10]. Similarly, numerous studies have established a positive association between high temperatures and bacterial gastroenteritis. A meta-analysis of 12 studies on temperature-related bacterial diarrhea identified a pooled incidence rate ratio of 1.07 [11]. More specifically, high temperatures are associated with elevated risks of shigellosis, *Escherichia* *coli* enteritis, salmonellosis, and cryptosporidiosis, while low temperatures are linked to increased risks of rotaviral and noroviral enteritis [3]. Although pathogen-specific analyses were not conducted in this study, evidence from Japan suggests a correlation between norovirus and rotavirus infections and colder temperatures, with lower incidence rates during summer months [12]. Additionally, an etiology-specific study conducted in South Korea from 2015 to 2019 found that temperature-pathogen relationships differ, with viruses more prevalent in colder conditions and bacteria more prevalent in warmer conditions, corroborating this study's findings [13]. These findings collectively suggest that temperature-related infectious gastroenteritis cases in Japan are predominantly driven by viral infections during winter months. However, additional etiology-specific evaluations are crucial for advancing future studies.

Our analysis also revealed that the MMT, representing the ambient temperature linked to the lowest risk of infectious gastroenteritis, varied across prefectures. The MMT functions as a key metric for evaluating the temperature-morbidity association, reflecting long-term climatic adaptation [14,15]. These variations likely stem from differences in socioeconomic factors, population demographics, geographic attributes, baseline disease risk, vulnerable groups, physiological adaptation, cultural practices, and regional weather conditions [14,15]. Further investigation is warranted to integrate these variables as meta-predictors. This finding emphasizes the necessity of in-depth research to assess the contributions of these factors to the observed burden in future analyses.

These findings underscore the critical need to elucidate how high and low temperatures influence the transmission of infectious gastroenteritis, enabling public health officials to identify factors driving temperature-related susceptibility and investigate variability in vulnerability. Such epidemiological insights can guide health authorities in enhancing projections of ambient temperature impacts on the infectious gastroenteritis burden. They also inform targeted public health strategies, including timely medical advisories, early warning systems, public health education, and optimized healthcare service preparedness.

To the best of our knowledge, this is the first nationwide study to quantify the morbidity due to infectious gastroenteritis attributable to cold and heat temperatures in Japan. However, this study has some limitations. First, we utilized an ecological study design. It is important to recognize that ecological studies can only partially control for the factors measured, leaving the possibility of uncontrolled confounding factors and residual bias. Second, we used meteorological data from fixed monitoring stations as surrogates of individual exposure, which may include measurement errors in exposure. Third, numerous factors beyond prefecture-specific temperature, temperature range, and relative humidity must be considered to explain the heterogeneity observed between prefectures. Future research incorporating demographic, economic, and health resource variables as meta-predictors would be valuable for a more comprehensive understanding. Fourth, since this study was conducted exclusively in Japan, caution should be exercised when generalizing the findings to other regions. Finally, our analysis was based on weekly data from 2000 to 2019. Extending the study period or utilizing a more detailed dataset could enhance the accuracy of these complex associations.

## Conclusions

In conclusion, this nationwide modeling study quantified a substantial burden of infectious gastroenteritis attributable to low ambient temperatures in Japan. This is one of the first nationwide studies in Japan to provide a quantitative assessment of morbidity risks linked to both cold and heat exposure, positioning the research as a significant contribution to the field. The findings highlight the necessity of integrating ambient temperature considerations into public health interventions aimed at mitigating the burden of infectious gastroenteritis. Moreover, localized public health strategies, adapted to the specific climatic conditions of individual prefectures, may enhance effectiveness in addressing temperature-related infectious gastroenteritis. Further research is warranted to advance understanding of the broader impacts of climate change on infectious disease patterns, particularly infectious gastroenteritis.
